# Audiovisual Self-Confrontation: Psychiatric and Psychotherapeutic Uses of Television and Video in (West) Germany 1970s–1990s

**DOI:** 10.1177/15274764241308830

**Published:** 2025-01-22

**Authors:** Renée Winter

**Affiliations:** 1 University of Vienna, Austria

**Keywords:** television in psychiatry, video therapy, self-confrontation, video feedback, mirroring, Internationaler Arbeitskreis für Audiovision in Psychiatrie und Psychotherapie

## Abstract

The history of television and video therapy in the Federal Republic of Germany is strongly linked to the working group IAAPP (Internationaler Arbeitskreis für Audiovision in Psychiatrie und Psychotherapie/International Working Group for Audiovision in Psychiatry and Psychotherapy), which was founded 1977 in West Berlin. Although a mainly German-speaking group, the IAAPP also regularly referred to studies from the U.S., but only selectively adopted their approaches concerning audiovisual practices in psychiatry. Technological and legal conditions for the implementation of television systems in psychiatric clinics were debated and elaborated by the working group and formed the basis for the development of various methods of television and video therapy. The members of the IAAPP were particularly interested in approaches to self-confrontation through video recordings which should induce self-reflection, compliance with therapeutic measures, or a coherent self-image.

## Introducing the Working Group: Internationaler Arbeitskreis für Audiovision in Psychiatrie und Psychotherapie

“In the application and study of psychiatric psychotherapy in the departments of general hospitals, video has become an indispensable medium.”^1^ (Ronge 1992, 191)

This statement was one of the results of a round table discussion at the thirteenth conference of the Internationaler Arbeitskreis für Audiovision in Psychiatrie und Psychotherapie/IAAPP (International Working Group for Audiovision in Psychiatry and Psychotherapy). The round table’s function was made explicit, namely, to provide arguments to justify the necessity of television and video recording systems to funding agencies and other stakeholders. Advocating for the use of audiovisual media was one of the tasks of the working group which was founded in 1977 in West Berlin. Starting from 1977 the IAAPP held eighteen annual conferences, fourteen of which (1977–1997) have been published in twelve conference volumes (Aebi et al. 1984; Hartwich 1999; Hartwich and Badura 1985; Helmchen and Renfordt 1978a; Kolitzus and Ellgring 1986; Kügelgen 1982a, 1989; Ronge 1980c, 1994, 1997; Ronge and Kügelgen 1992; Stille and Hartwich 1983). In the founding years, there existed a journal for members of the working group “Video-Informationen” which was used to communicate in between the annual meetings. Speakers at the conferences were mainly psychiatrists, psychotherapists, and technicians. Also involved were other professional groups working in psychiatric hospitals, such as psychologists or social workers and social pedagogues.

Using the example of the IAAPP, the article traces the introduction of video technology in psychiatric clinics in West Germany. To this end, it examines the spatial arrangements, the legal level, as well as specific therapeutic implementations. The article also asks which concepts and publications the West German psychiatrists did (not) refer to in their use of video technology to assess the assumptions informing the introduction of video. The focus is particularly on practices of self-confrontation, with an interest in underlying conceptions about illness and health, normality and deviance, and power relations within the clinics.

Video entered West German psychiatry at a time when reforms were intensively discussed and implemented over decades (Brink 2010, 410–493; Dörre 2021, 425–494), which also meant a departure from large, closed institutions (Rudloff et al. 2022). Social psychiatry was (in contrast to antipsychiatry, as I will show later) a relevant field for some, but not for the majority of the members of the IAAPP.^2^ Engaging in social psychiatric discourses meant that both in terms of causes and treatment of mental health disorders, not only biological and neurological explanations but also social and cultural factors were considered relevant. Although the relationship between social psychiatry and antipsychiatry in the 1960s and 1970s was complicated because social psychiatrists distanced themselves from antipsychiatry (Brink 2010, 456–457) while antipsychiatry activists criticized social psychiatry for not being radical enough (Dörre 2021, 429), the engagement of social psychiatrists within the working group meant that some of the central actors of the IAAPP were interested in changes of psychiatric institutions.

Early aims of the working group were that an “inventory of the television equipment available in psychiatric, psychotherapeutic and clinical-psychological institutions” (Köhler and Miller 1989, 3–4) should be made, mutual information on the technical possibilities, use and methods should be encouraged and that the working group should explicitly include “all natural or legal persons who make use of audiovisual procedures” (6–7). Some of the early goals were very ambitious and could not be met for example the establishment of a nationwide video library (Cording 1980; Kügelgen and Penning 1982, 124) or to carry out own research programs by the working group (Köhler and Miller 1989, 8). Another goal was to introduce a new job profile – the so-called video assistant –, to obtain its governmental recognition and to be involved in the development of corresponding educational institutions to train these assistants (Cording 1980; Köhler and Miller 1980; Ronge 1980b). Video assistants or people who had the technological know-how and the knowledge about how psychiatric clinics or psychotherapy worked were highly sought-after and much needed in the first years of the working group around 1980. With the popularization of video technology, the further development of consumer formats, and the increasing ease of operating the devices during the 1980s, the demand for highly specialized labor in this field apparently decreased.

With regard to methods and approaches, the working group was concerned with audiovisual practices in documentation, diagnostics, teaching, research and therapy. Concrete usage scenarios (beyond uses in therapy on which I will elaborate later) that were discussed in the working group included for example television and video in education and training. Here, the argument was made for the protection of patients, as they would no longer have to appear directly in the lecture hall before the students. At the same time, video recordings were used for self-reflection by students when they were being observed during observation (Schepank 1980). TV systems in the clinics were used for surveillance purposes, such as in the case of the “electronic sitting watch.” This process was primarily justified on economic grounds, as it made it possible for one person to monitor multiple patients simultaneously (Ronge 1982, 1989). And video was in manifold ways deployed to enhance diagnostics, documentation, and research. It was used to improve ratings, to make patients, symptoms and conditions measurable and quantifiable, to examine the effects of psychotropic medications, or to document doctor-patient-conversations, symptoms and self-assessments.

In their contribution for the 1980 annual conference, Gert-Klaus Köhler and Marianne Miller identified twenty-two sites where video was being used in psychiatry and psychotherapy, partly connected to the working group (Köhler and Miller 1982, 39). The “international” aspect of the working group was limited, it referred primarily to various countries in the German-speaking world. The majority of members and contributors came from and worked in the Federal Republic of Germany and to a lesser extent Switzerland. Austrian psychiatrists participated at least in two conferences (Katschnig and Wanschura 1992; Meise et al. 1994; Springer-Kremser 1992), studies from psychiatrists of the GDR were only rarely mentioned.^3^ Despite this very limited international focus, U.S. studies were regularly referenced and discussed.

## Selective References to U.S. Debates on Psychiatric Uses of Television and Video

**Figure 1.**
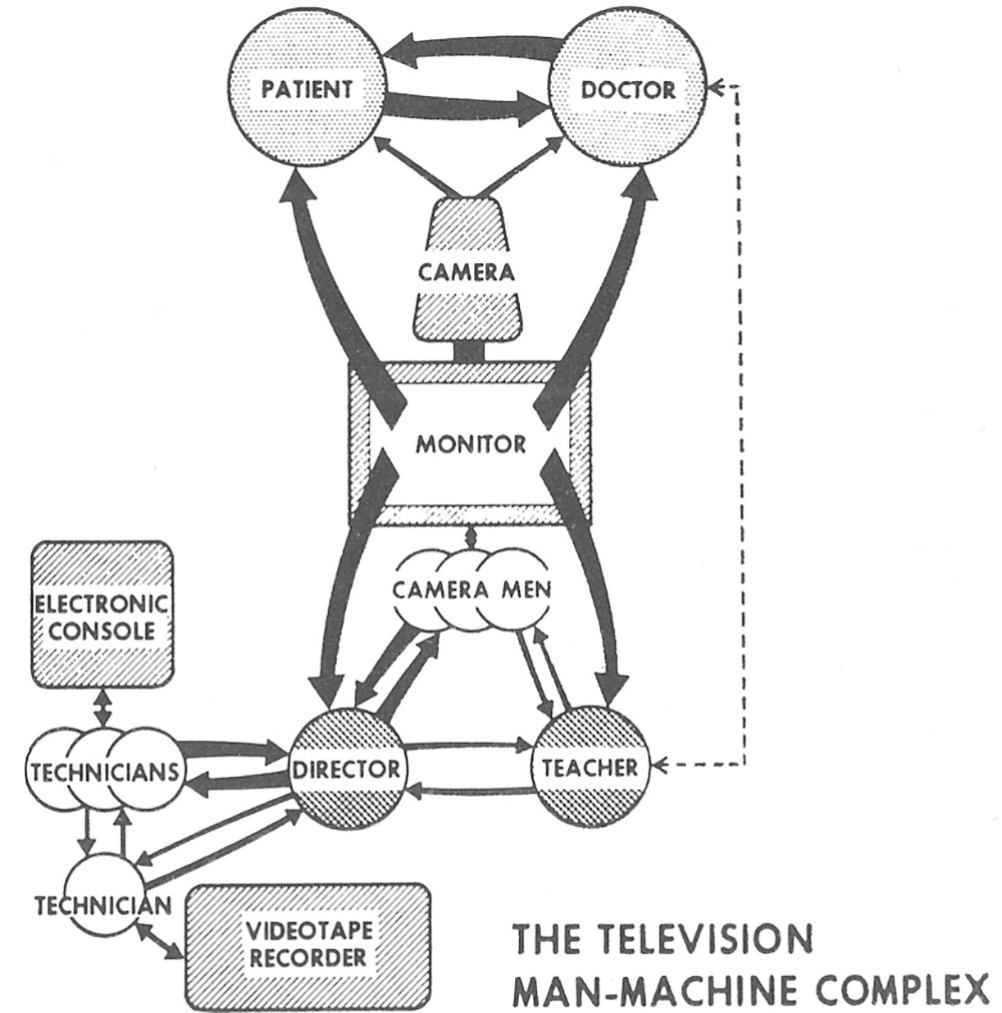
“The television man-machine complex.” (Wilmer 1970, 216).

The use of television and video in psychotherapy and psychiatry became a lively debated topic in the academic and professional discourse in the U.S. in the mid-1960s. In 1963 Norman Kagan and others began to publish several articles about a video-assisted method of therapeutic self-reflection called “Interpersonal process recall” (IPR; Kagan et al. 1963). For training and education purposes a video series was produced by Michigan State University, recorded in the 1970s and published later as a DVD collection in 1995. As was emphasized, the use of video should lead to a more balanced relationship, the relationship between therapist and client should become a “professional partnership” and the client should emerge empowered from the audiovisual confrontation.^4^ In their article on “The Use of Videotape Recordings in Conjoint Marital Therapy”, Alger and Hogan (1967) expressed the very hopeful and enthusiastic estimation that “videotape recording represents a technological breakthrough with the kind of significance for psychiatry that the microscope has had for biology.” (1,425) This statement has then been picked up or quoted very often (see also Köhler and Miller 1982, 33), for example by Berger (1973) in the activist and artist journal *Radical Software*, published in New York City from 1970 to 1974. Milton Berger was a psychiatrist and video enthusiast, he had the position of Assistant Clinical Professor of Psychiatry at the College of Physicians and Surgeons, Columbia University when he published a much-received anthology on “Videotape Techniques in Psychiatric Training and Treatment” in Berger (1970). The anthology brought together contributions on various methods of training and therapy, on legal conditions and technical issues. The fact that Milton Berger also wrote for *Radical Software*, and that the anthology was referred to in the journal (Radical Software 1971: 9), demonstrates the close connection between early video use in psychiatry in the United States and activist contexts and discourses – a connection that historian Peter Sachs Collopy (2015) has extensively examined in his study on video “as a technology of consciousness in the long 1960s.” One linking element was cybernetic discourses, which were adopted by both video artists and activists, as well as in psychiatry. Cybernetics allowed for the theoretical exploration and reimagining of the relationship between (video) technology and human subjects. Cyberneticians who were well-received in the context of *Radical Software* (like e.g., Gregory Bateson) offered new theoretical frameworks for understanding the self and psychiatric diagnoses like schizophrenia. Geoghegan (2017) described this interplay of media technology, psychotherapy, and cybernetics through the Palo Alto Group in the 1950s and 1960s. Though the group primarily operated with film and audio tape, their work laid the foundation for a later direction of video therapy. Geoghegan argues that the group produced “the family as cybernetic machine,” as “a functioning media technology composed of film strips, feedback loops, photographs, studios, audio recordings, mirrors, mothers, fathers, children, and therapists” (71–72). A similar idea of functioning and human-machine interaction is visualized in a figure titled “the television man-machine complex” in the abovementioned anthology by Milton Berger (see Figure 1). The figure is part of the contribution by Wilmer (1970, 216), who was a Clinical Professor of Psychiatry at the University of California at the time. In his text “Technical and Artistic Aspects of Videotape in Psychiatric Teaching,” Wilmer described video feedback methods, supported by split screens and superimpositions. The diagram served him to show the skills required by the involved participants, namely, to make quick decisions based on interlocking workflows: “The team should resemble the surgical theater with its quiet, quickly responsive, mutual intent on the serious work at hand.” (216) In Wilmer’s schema, technical devices, technicians, patients, and doctors are equally interacting elements of a complex ensemble.

As Grimaldi (2017) has shown, Harry Wilmer’s intensive engagement with the possibilities of video feedback in psychiatry emerged in the context of his critical attitude toward his own profession. Wilmer was considered anti-authoritarian and revolutionary: “He liked to think of himself as the heir to Philippe Pinel, the famous doctor of the French Revolution, who unchained his patients and aspired to cure them through reasoned conversation.” (99)

As I would like to demonstrate, the implementation of video and television in psychiatry in the Federal Republic of Germany (BRD) had a different history. It was not activists and only rarely psychiatrists aiming to radically change psychiatry who introduced video into psychiatric clinics, but to a large part psychiatrists who aimed to improve diagnoses, assessments, education, research, and therapies. Although Milton Berger’s anthology and other papers from the U.S. context have been acknowledged and cited in contributions of the IAAPP, it appears that they may have lost their critical context during the process of adoption.

While in the U.S. early video use in psychiatry was partly linked to antipsychiatric discourses or at least a critique of dominant practices of psychiatry, antipsychiatry and critical voices towards psychiatry were discourses from which the predominantly German members of the IAAPP strongly distanced themselves. The topic usually only came up in regard to film and television productions about psychiatry that were suspected to be spreading a false image (Cording-Tömmel 1986; Kolitzus 1984, 1986; Tretter 1986, 1989) whereas video was seen as a medium by doctors and heads of institutions that could be used to counter these “distorted images by the media” with one’s own perspectives (Köhler and Marianne 1983; Meise et al. 1994).

Thus, the working group also remained within the general framework of German academic psychiatry, for which Benoît Majerus (2008, 349) noted that historically (in contrast to, e.g., the Netherlands or Italy), “hardly any points of contact between academic psychiatry and antipsychiatry” can be identified.

## Technological Arrangements: On (Not) Hiding and (Not) Disturbing

Film as a recording medium barely played a role for the IAAPP, video was preferred for a number of reasons, its long runtime, the cheapness of the material, which mainly supported rationalization arguments, the immediacy (no development time), and the easier usability “for technical and artistic novices, such as a doctor” (Kügelgen and Penning 1982, 123).

In the 1970s the clinics worked mainly with 1 in and 2 in videotapes; very soon other video formats came into focus. Clemens Cording (1980), psychiatrist at the Max Planck Institute for Psychiatry in Munich, proclaimed the advantages of VHS at the 1979 annual conference: in a paper with the programmatic title *Economy instead of Perfection*, he argued that VHS was cheaper, less complex, easier to operate and less susceptible to interference. Johann Heinrich Ellgring (1983), who also worked at the Max Planck Institute in Munich, complained in 1981 that the available formats, U-matic, VHS, VCR and Betamax were not compatible, which led to problems with tape exchange. These early discussions about formats that were very similar to those held in activist video groups, were humorously addressed in the members’ magazine Video-Informationen (1980/81, 14).

Descriptions of the technical facilities make up a large part of the early conference proceedings. The audiovisual department of the Psychiatric Clinic of the Free University (FU) of Berlin is very well documented, partly because its director, Ernst Renfordt, was the first president of the IAAPP (Helmchen 1980, 62). Ernst Renfordt, born in 1937, had studied medicine in Münster and West Berlin, and obtained his doctorate in 1971 at the FU Berlin in the field of psychiatry.^5^ He began setting up an audiovisual system at the psychiatric clinic of the FU Berlin in 1970 and from then on, together with the technician Hartmut Kluge, he supervised the development of video technology in the psychiatric department (Helmchen 1975, 27).

During the relocation and new construction of the clinic from 1976 to 1979, the requirements of the audiovisual department were considered right from the start. The clinic now had a “control center, two TV studios with a total of 6 remotely controllable cameras, a workshop, an office, and the video archive room.” (Helmchen 1980, 32; see also Helmchen 1985, 41–42; Helmchen 1990, 72) However, the ideal recording situation and the ideal setting for therapeutic or other clinical purposes contradicted each other, as Kluge and Renfordt (1980, 233) acknowledged:

The television facilities and the television recording conditions in psychiatry and psychotherapy must be designed in such a way that 1. the doctor-patient situation remains undisturbed whenever possible and that 2. the process of recording, for ethical and psychological reasons, is not concealed from either the doctor or the patient. Both conditions seem to contradict each other in that the first aspect leans more towards concealment, while the second aspect leans more towards revealing the technical instrumentation.

The technical facilities therefore had to be designed in such a way that these contradictions were balanced. Since both cameras and operating cameramen could interfere with the intimacy of the therapy situation (Kluge and Renfordt 1978, 87f.;^1980^, 235), remote-controlled cameras were placed in boxes that were integrated into the furniture, painted black on the in- and outside and covered with a windowpane to eliminate “visual and audio attention-triggering signals,” not totally hiding, but also not exposing them (Kluge and Renfordt 1980, 236).^6^

**Figure 2.**
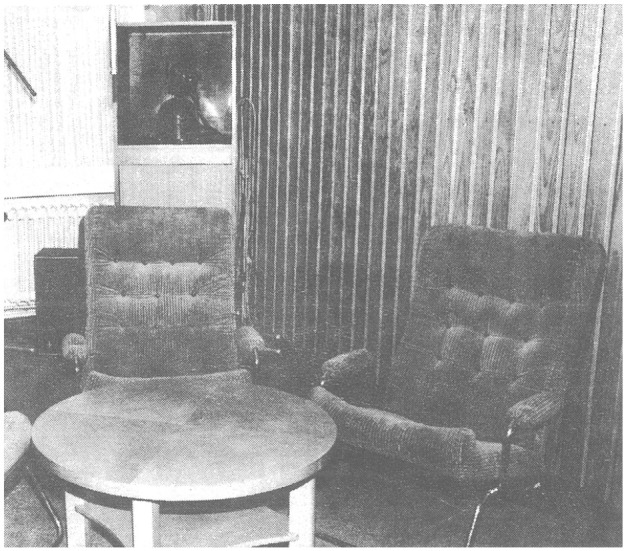
TV recording studio with a remote-controlled camera in the background. (Janssen 1982: 22).

Janssen (1982, 22) published a photo (see Figure 2) showing one of these camera boxes in the TV studio of the clinic of the FU Berlin. In her dissertation, Janssen explored psychiatric patients’ reactions to television recordings. While Janssen aimed to identify and possibly reduce potential disruptions in patient-doctor interaction or other influences of the recording on the situation, her study unintentionally provides valuable insight into patients’ perspectives, into the expectations and fears of patients regarding video recordings, and the resistances and rejections toward the recordings.

## Legal Discussions and Knowledge Distribution: On (Not) Showing Records to Patients

Discussions about the legal framework also took up a lot of space in the meetings of the first few years. In particular, the regulations on medical confidentiality and data protection limited the possibilities of filming videos. In the first conference volume, 1978, various problems were raised in obtaining the consent of the patients to pass on the audiovisual material or to release the psychiatrist from the obligation of confidentiality (Helmchen and Renfordt 1978b). This consent was the prerequisite for the doctors to use the videotapes for teaching, research, and other purposes.^7^ In a panel discussion, the question was raised as to whether the patient needed to know the material that they were giving consent to be shared. Ernst Renfordt, together with Hanfried Helmchen, then director of the psychiatric department at the FU Berlin, summarized the answer to this question as follows:

“Even if the patient, according to our constitution [. . .], has a right to information about his illness and also about all the data of his medical history, a doctor will as a rule only inform him to the extent that this is possible without harm to the patient after weighing the interests of the patient. The same is certainly true for the content of a medical history, which, by the way, can hardly be made accessible to the patient, because the doctor could lose the independence of his judgment if he always had to keep his records in mind that the patient might read them one day. Also, a medical record is a physician’s record *about* the patient, whereas an audiovisual record is a direct document of medical report, perhaps more comparable to an x-ray image. But if one takes into account the wrong conclusions some patients already draw from their x-rays, then the serious medical reservations about confronting the patient [. . .] with an av record of his behavior during the illness, which is incomprehensible or frightening to him, become understandable.” (Helmchen and Renfordt 1978b, 83)

While it is argued here first with the protection of the patient, it becomes evident that one important concern is to prevent the psychiatrist from being put in the position of writing the records in such a way that they can be read by the patient. What becomes obvious is that the patients should remain the object of the knowledge produced about them (and not knowing subjects). They are not seen as capable of correctly interpreting the data produced about their body and psyche.^8^

The legal framework – in combination with semi-hidden cameras in the psychiatric clinics – is involved here in creating a panoptic situation in the Foucauldian sense, in which knowledge and seeing or the possibility of insight (into the audiovisual or medical records) are unequally distributed. However, patients were able to see their recordings in a controlled and selective way, they even *should* watch them for therapeutic reasons in audiovisual self-confrontation.

## Self-Confrontation With Video: Video Mirroring

**Figure 3.**
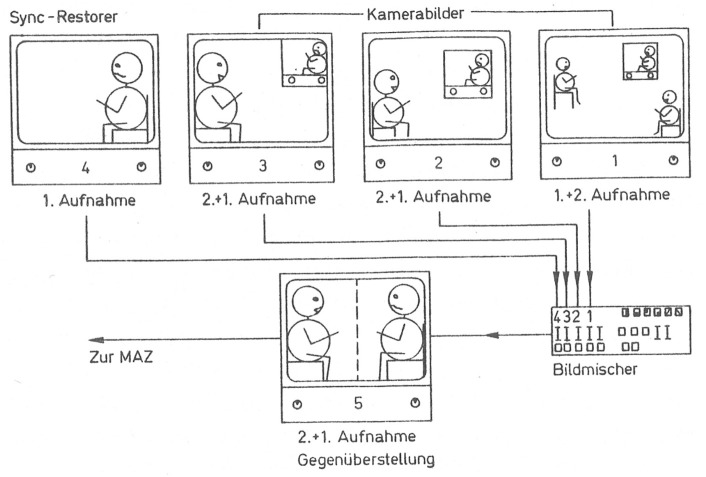
“On Monitor 1, the overall situation with the patient and the doctor viewing the initial recording is recognizable. Monitors 2 and 3 display a modification of the overview recording. Monitor 4 shows a segment from the archived video recording of the patient, to which the current recording (Monitor 5) is added.” (Ronge 1980a, 197).

One attraction of therapeutic work with video for the psychiatrists of the IAAPP lay in the possibility of immediate feedback to the patients. Many contributions focused on methods of therapy through “self-confrontation,” “video mirroring,” or “video feedback.” Although there are semantic differences between these terms, they were largely used synonymously. Ronge (1980a,1980b) provided rather general descriptions of the technical arrangement and the procedure of video confrontation (see Figure 3); most psychiatrists discussed specific applications within psychiatry. In the following, I would like to briefly outline how the treatment of various disorders with video feedback should work according to the psychiatrists and what functions have been attributed to the medium in this context. I do this on the example of main areas in which video feedback was used: group, couple or family therapy; the treatment of eating disorders as well as drug or alcohol abuse, and the treatment of schizophrenia.

Group, couple or family therapy were early areas of employment of video technology, where the predominantly German authors could refer to a range of experiences – especially from psychiatrists in the U.S. The members of the working group emphasized many positive effects of video in therapy: an improvement in “perception and change of social interaction” since video allows “to observe one’s own behavior critically,” as Ellgring (1982, 214) stated. In self-modeling – “learning from one’s own model,” desired behaviors are demonstrated to the patient by themself on video – supposedly achieving “an intensive impression” (Ellgring 1982, 216). Lehmkuhl and Bonney (1984, 109) emphasized that video allows “immediate and objective feedback” since “the immediacy of the recording eliminates the danger of forgetting, repression or misinterpretation,” “video feedback clarifies self-image and other-image discrepancies” and “facilitates the process of self-awareness, it provides a mirror for one’s own behavior and experience” (see also Lehmkuhl and Eisert 1983, 160). It thereby could improve self-perception and relationships (Bonney and Lehmkuhl 1984, 119). However, these lists of positive effects were accompanied by limitations. It was pointed out that confronting patients with their misconduct could have negative effects on self-perception (Ellgring 1982, 215) and there were also skeptic voices towards its use in therapy (218–219). For example, psychiatrists Lehmkuhl and Seeger (1992, 121), who emphasized that the “effect of self-confrontation in group psychotherapy lies, according to Danet (1968), in the experience of immediate repeated experience of oneself and in the additional experience of the individual being able to see how others perceive him in the group” (121), acknowledged that video feedback at the beginning “can be a good introduction [. . .] but later reinforces defensive behavior and resistance.” (122) In the case of severe personality disorders and acute psychoses, video should also be avoided. (Lehmkuhl and Seeger 1992, 123–124)

The methods of video use for anorexia and adiposity as well as for alcohol and drug abuse were closely linked, and behavioral therapy approaches dominated the field: the wrong behavior was to be unlearned through video therapy. Video confrontation should promote acceptance of the disease (*Krankheitseinsicht*; Badura and Heizmann 1983, 100; see also Luderer and Böcker 1984; Hartwich and Schumacher 1984) which should be achieved, for example, by showing the patients recordings in which they can be seen drunk, resulting in a “shock effect” (Ellgring 1982, 214).

Also, in the case of anorexia or adiposity, a profound encounter with one’s own appearance by means of video was insisted upon by the doctors (Badura and Heizmann 1983; Brüggemann 1984; Ellgring 1982, 1983; Hartwich and Schumacher 1984; Krahl and Meermann 1984; Meermann 1985; Napierski et al. 1985). The descriptions of the procedures paint a picture of an arrangement of bodies, gazes, and technology shaped by power dynamics.^9^ The patients, most of them women, were in swimsuits or gym clothes in the video studio exposed to the gaze of the predominantly male psychiatrists, video technicians, and cameras (Krahl and Meermann 1984; Meermann 1985, 50–51; 55–57; 59–60). It was recommended to zoom in on certain parts of the body. For the presentation of the videos and the confrontation of the patients, Meermann (1985, 57) recommended a high quality and color reproduction on a scale of 1:1. Here, it was also acknowledged that recordings could be potentially distressing for patients (Lehmkuhl and Seeger 1992, 119). Brüggemann (1984, 8) reports that one patient quit video confrontation therapy because she felt “other-directed and misunderstood.”^10^

Self-confrontation in the treatment of schizophrenia was dealt with mainly by Peter Hartwich. Hartwich, born in 1940 in Dresden (Hartwich 1965, 68), was a psychiatrist in Aachen before becoming head of the psychiatric clinic in Frankfurt am Main. He published extensively on audiovisual self-confrontation and schizophrenia and was the last president of the IAAPP. Against the background that few significant studies were confirming the effectiveness of self-confrontation at the beginning of the 1980s, Hartwich’s publications were primarily concerned with empirically proving the effects of video therapy through various experimental set-ups. The results of his calculations were that only in a few cases could effectiveness be proven and that the positive effect was only verifiable for a short period (Hartwich and Lehmkuhl 1980, 31; Hartwich 1982, 246; Hartwich and Deister 1983, 52–54). In the mid-1980s, he published a classification of personality structures and psychopathological diagnoses concerning the indication and contraindication of video confrontation. Although Hartwich (1984, 38) concluded here that there are several contraindications and that video therapy should be used only in selected cases and then only as an additional means – also his student Deister (1985, 141, 143) saw an overestimation of video therapy and video technology^11^ – subsequent publications again emphasized above all the advantages of video therapy. Thus in 1992, Hartwich described as possible effects: “improvement of a feeling of coherence” (Hartwich 1992, 74), “reduction of cognitive impairment” (77), “enhancement of affective capacity” (78), and “stimulation of motivation and drive” (80). He continued to focus on specific effects of video confrontation on the self, linking psychiatric research and the cultural history of the mirror. One of his main theses was that schizophrenia causes a fragmentation of the self that could be reversed by video mirroring, thereby restoring a coherent self (Hartwich 2018).

This function of video is built on the assumption that the medium could record an objective reality (a unified self, not a fragmented one) that would then be perceived accordingly by the patients. An anecdote from Peter Hartwich in an interview with me (2 May 2022) shows the possible failure of the evidentiary function of video recordings in therapy: Hartwich reported about a patient who complained that the look of her eyes had changed so severely and that they were so strange to her. The patient was shown a video of herself to show her that her eyes had not changed at all and that she looked “completely normal.” Her response to the recording was “Look there, doctor, this is exactly what I wanted to show you.”

As the examples have shown, the supposed functions of seeing oneself in the video were dependent on the context. Approaches of group and family therapy assumed that seeing oneself in social interaction can initiate and facilitate reflection on self-perception and relationships, thus via the medium the subject should reflect themself. In behavioral therapy approaches to eating disorders and alcohol abuse, the medium was intended to induce insight into one’s behavior. Via the medium the subject should accept themself as not healthy and/or suffering in order to achieve compliance to therapeutic measures. Video confrontation here should lead to self-awareness as ill, sometimes by trying to induce shame in the patients. This approach can be linked back to an enlightened approach to madness. Foucault (1988, 262–263) described how the chains detaining those described as mad were replaced by other technologies, one of them the mirror, in which the person defined as mad was forced to recognize themself as mad. In the context of a schizophrenia diagnosis, video, in turn, was supposed to lead to self-acceptance as coherent.

In reference to the panoptic gaze dispositive described by Michel Foucault, Rozenkrantz (2018, 2020, 93–110) has described the U.S. use of video feedback in therapy as an “autopticon,” through which the patients are encouraged to look at themselves as others would see them, implying a gaze “oscillating between self-correction and self-acceptance” (2020, 116), positioning the patient as their own supervisor (108). He emphasizes that this presupposed gaze is always a historically situated one (105–106).

The failures of this autoptic dispositive are evident not only in the lack of adoption of the gaze, as suggested in the anecdote above, but also in many small resistances documented by the authors, such as painting over the camera lens (Ronge 1989, 85), discontinuing therapy (Brüggemann 1984, 8), or expressing dissatisfaction with the recording situation (Janssen 1982, 64–67).

## Conclusion: Turning the Mirror

Joachim Ronge (1980a), psychiatrist in Ludwigsburg, explained the positive effects of confronting recovered patients with their earlier recordings. He was convinced that audiovisual confrontation could strengthen the motivation to take medication or accept other measures and could also encourage relatives to cooperate; in short, it could help achieve compliance. (202). One function of television and video in psychiatry was, therefore, to ensure the smooth operation of the processes in the clinic. Other promises of the medium concerned the improvement of diagnostics, teaching, documentation, and therapy. The publications of the IAAPP indicate a strong demand and a strong will to record as much as possible. The psychiatrists involved wanted to produce, accumulate, and share audiovisual knowledge. Technology and law provided the regulatory framework to make this ambition for successful video recording of patients a reality. Ethical considerations and the protection of patients were discussed but also instrumentalized insofar as they were intended to stabilize an unequal distribution of (audiovisual) knowledge.

With the new media, new actors entered the field, technicians, and companies, some of which also financed the activities of the working group.^12^ Video was used as an epistemological tool and linked to relatively new but already existing methods, such as the uses of psychotropic drugs in therapy, the effects of which could be better studied with the help of video recordings. Video was also seen and used as a PR tool and in various ways in therapy. The medium could optimize surveillance measures and improve education and training. As I have shown, in West German psychiatry television and video were not primarily introduced in a critical approach to existing structures and hierarchies in psychiatry. However, within the working group, neurology-oriented psychiatrists worked together with social psychiatrists and psychotherapists.

The observations in this article are mainly based on sources from the perspective of psychiatrists and psychotherapists. Sources from the patients’ perspective are rare, and if they are available in archives, they are often not (yet) accessible for data protection reasons. I have tried to trace some of these perspectives by reading the psychiatric texts against the grain and to pay attention to the patients’ acts of resistances to being filmed.

I would like to conclude this article with a note on the self-organization of people with psychiatric experience that was taking place in West Germany in the 1980s. The gaze dispositive, of which the video cameras in the psychiatric clinics were a part, was taken up and politically turned by the Irren-Offensive (Mad Offensive), founded in 1981. At the same time that psychiatrists were discussing the use of audiovisual self-confrontation, the Irren-Offensive, an activist self-organization of psychiatric survivors, as they called themselves, was founded in West Berlin. Stöckle (2005), a member of the group, conducted interviews with other members about their psychiatric experiences in the early 1980s. Video did not play a role in these memories, but a mirror did. At an exhibition on open psychiatry in Trieste “Bestandsaufnahme einer Psychiatrie, Wahnsinn und Ausgrenzung in Fotodokumenten” (Taking stock of a psychiatry, madness and exclusion in photographic documents), in 1981 in West Berlin, one contribution of the Irren-Offensive was to put up a mirror “which enables the compulsively normal to also view themselves within the exhibition” (281). In a later interview, the activist Bruckmann (1993, 377) remembered “I like to think back to the mirror at the Basaglia exhibition, above which hung the ironic text: ‘Ever seen someone obsessively normal?’ There, the curious exhibition visitors could observe themselves. It gave me great joy how upbeat we were – unbeatable.”

The mirror was used by the activists to confront the observers. While this intervention criticized the exhibition’s representational practices, it also exposed the call for self-confrontation as a potential strategy of othering.

Finally, the idea of turning the mirror can also be applied to the media and mental health discourse. Beyond the question of how media can be beneficial (or threatening) to mental health, by analyzing how video was inscribed in psychiatric and psychotherapeutic constellations of power and orders of knowledge, the article aims to contribute to historicizing the relationship between psychiatry and media without assuming either of them a priori.

## Notes

1. All translations are by the author.

2. Peter Hartwich translated the work of the social psychiatrist John K. Wing, titled “Reasoning about Madness,” which was originally published in English in 1978, into German (Wing John 1982). Heinz Häfner, one of the earliest proponents of social psychiatry in West Germany (see Dörre 2021, 422–423; 428), wrote in the conference proceedings of 1992 (Häfner et al. 1992). In 1984, the conference took place, among other locations, at the Social Psychiatric University Clinic in Bern (Aebi et al. 1984) and the publication of the conference proceedings was partially organized in collaboration with social psychiatric institutions (see e.g., Stille and Hartwich 1983).

3. Köhler and Miller (1982) cited the work of Kühne et al. (1978) who worked on television in psychiatry in Magdeburg.

4. See the concluding statement by Henya Kagan (Klein), in the Video on “The Recall Process - Individual and Mutual Recall” (#IPR F-3). The term “client,” used here by Henya Kagan, is less tied to a medical model of illness than the term “patient.” In the German-language publications I analyzed, the term “patient” is predominantly used, even in sources from anti-psychiatry activists.

5. Archive of the FU Berlin: Universitätsarchiv: Studentenakte: Stud A 45,843 Promotionsakte: Prom-HMed, 3,284.

6. Other conflicting issues were light (uncontrollable daylight vs. the unpleasant atmosphere of windowless rooms) and sound (disturbing microphones, and the difficulties to reduce excessive reverberation without over-damping). (Kluge and Renfordt 1980, 235–238)

7. Kügelgen (1982b, 158) published an example of how patients were informed to get their consent.

8. In 1980, two years later, the IAAPP published recommendations for the creation and use of television recordings of psychiatric patients. While these recommendations generally support the patient’s right to access the recordings, they also refer to the potential harm that insights into the recordings can cause: “Upon request, the recording should be shown to the patient if, from a medical-therapeutic perspective, this can be done without harm to the patient.” (Renfordt 1980, 231). The debates over patients’ access to the records can also be seen in the context of a court proceeding that took place shortly thereafter. Peter Lehmann, educator, antipsychiatry activist and former patient of Hanfried Helmchen, demanded to get access to his own medical records which Helmchen denied him. The court ruled in favor of Peter Lehmann (Helmchen 2007, 114f; Lehmann 2023). In the course of the ensuing court case in 1982, the historian Aly (1982, 9) also wrote an article in the TAZ in which he pointed out Helmchen’s problematic methods and “human experiments” (mainly referring to uses of neuroleptics and electric shock therapy).

9. How deeply these practices were inscribed in gendered power structures is demonstrated by the public disclosure of one of the authors decades later. Walter Vandereycken, psychiatrist and university professor in Belgium, who specialized in eating disorders, was revealed in 2012 to have had non-consensual sexual contact with female patients over the last twenty years and was suspended (BRF Nachrichten 2012).

10. Brüggemann (1984) retrospectively acknowledged that there would have been an alternative to video confrontation therapy, namely the approach of the 1978 published book “Fat is a feminist issue” by Susie Orbach.

11. See also Gagel (1994, 107), who described the disadvantages and dangers such as the destabilization of the patient.

12. Levy-Landesberg and Pinchevski (2023) have described a similar process with the introduction of audio recordings into psychotherapy. They explain how “the therapeutic session became an apparatus [. . .], encompassing control room technicians, stenographers, research assistants, and outside colleagues, supervisors, and students.” (132)
